# Functional Impact of Corticotropin-Releasing Factor Exposure on Tau Phosphorylation and Axon Transport

**DOI:** 10.1371/journal.pone.0147250

**Published:** 2016-01-20

**Authors:** Michelle H. Le, April M. Weissmiller, Louise Monte, Po Han Lin, Tia C. Hexom, Orlangie Natera, Chengbiao Wu, Robert A. Rissman

**Affiliations:** 1 Department of Neurosciences, University of California San Diego, La Jolla, CA 92093, United States of America; 2 Veterans Affairs San Diego Healthcare System, San Diego, CA 92161, United States of America; Oregon Health and Science University, UNITED STATES

## Abstract

Stress exposure or increased levels of corticotropin-releasing factor (CRF) induce hippocampal tau phosphorylation (tau-P) in rodent models, a process that is dependent on the type-1 CRF receptor (CRFR1). Although these preclinical studies on stress-induced tau-P provide mechanistic insight for epidemiological work that identifies stress as a risk factor for Alzheimer’s disease (AD), the actual impact of stress-induced tau-P on neuronal function remains unclear. To determine the functional consequences of stress-induced tau-P, we developed a novel mouse neuronal cell culture system to explore the impact of acute (0.5hr) and chronic (2hr) CRF treatment on tau-P and integral cell processes such as axon transport. Consistent with in vivo reports, we found that chronic CRF treatment increased tau-P levels and caused globular accumulations of phosphorylated tau in dendritic and axonal processes. Furthermore, while both acute and chronic CRF treatment led to significant reduction in CREB activation and axon transport of brain-derived neurotrophic factor (BDNF), this was not the case with mitochondrial transport. Acute CRF treatment caused increased mitochondrial velocity and distance traveled in neurons, while chronic CRF treatment modestly decreased mitochondrial velocity and greatly increased distance traveled. These results suggest that transport of cellular energetics may take priority over growth factors during stress. Tau-P was required for these changes, as co-treatment of CRF with a GSK kinase inhibitor prevented CRF-induced tau-P and all axon transport changes. Collectively, our results provide mechanistic insight into the consequences of stress peptide-induced tau-P and provide an explanation for how chronic stress via CRF may lead to neuronal vulnerability in AD.

## Introduction

Alzheimer’s disease (AD) is dementia disorder characterized by extensive synaptic and neuronal loss, extracellular amyloid beta (Aβ) plaques and intracellular neurofibrillary tangles (NFTs). The accumulation of Aβ plaques is hypothesized to precede dementia in AD (for review, [1]), while synaptic loss and NFT accumulation are thought to occur later and have been found to directly correlate with worsening cognitive impairment [2–4].

NFTs are composed of hyperphosphorylated and aggregated forms of the microtubule associated protein, tau. Tau is a soluble phospho-protein that exists in multiple isoforms and plays an important role in stabilizing microtubules and in the maintenance of neuronal structure, polarity and axon transport [5–12]. Although some information is known about hyperphosphorylated tau in AD NFTs [9, 13–15], the precise mechanistic role that tau phosphorylation (tau-P) plays in neuronal compromise has been difficult to pinpoint. One hypothesis in the field suggests that excessive or sustained levels of phosphorylation reduce the ability of tau to bind and stabilize microtubules, leading to tau aggregation, impaired axonal transport and eventually NFTs [16–20].

AD is predominantly a sporadic disease, with less than 2% of cases linked to specific genetic mutations. An extensive literature implicates chronic stress in the development of sporadic AD [21–25] which supports a body of epidemiological work demonstrating that individuals prone to experience psychological distress or anxiety have accelerated rates of cognitive decline and are three times more likely to be diagnosed with AD [26–29]. Furthermore, stress exposure in humans affects learning, memory, hippocampal function and morphology [26, 27].

Although corticotropin-releasing factor (CRF) is best recognized as the hypothalamic neuropeptide that governs the endocrine stress response [30, 31], its distribution and actions in the CNS are similar to that of a neuromodulator/neurotransmitter [32–34]. The CRF family of peptides exert their biological effects via two G protein-coupled receptors (CRFRs) that are positively coupled to adenylate cyclase. CRF binds CRFR1 with high affinity, and in the pituitary gland this interaction mediates the neuroendocrine stress response [35]. CRFR1 is widely expressed in the brain, including AD-relevant areas such as the neocortex and hippocampus [36]. CRFR2 is a structurally related receptor but displays very limited CNS distribution [37]. The low affinity of CRF for CRFR2 led to the identification of three additional members of the CRF family, the urocortin (UCN) peptides. UCN 2 and 3 serve as high affinity CRFR2-selective ligands, and UCN 1 binds both receptors with comparably high affinities [38–40].

Supporting the hypothesis of broad central activity of CRF, studies have found prominent changes in the CRF signaling system in brain areas that are vulnerable to AD neuropathology and cell loss [41–43]. In addition to the CRF peptide itself, considerable attention has been focused on stress steroids (e.g. cortisol, corticosterone), effectors of the stress cascade, as mediators of neuronal vulnerability in AD. Increased circulating levels of cortisol in aged individuals has been linked to brain atrophy and a range of adverse effects in the hippocampus [21, 44–46]. Furthermore, results from clinical trials suggest that corticosteroid treatment worsens behavioral symptoms in AD patients [47]. Taken together, these human studies suggest that alternations occur in CRF/stress circuitry in AD and that these changes may directly impact AD-symptomology and neuropathology.

Work in AD models also supports a role for stress in disease pathogenesis. For example, many AD mouse models display increased anxiety behavior and perturbations in central stress signaling [48–51]. With regard to AD tau neuropathology, an abundance of rodent studies demonstrate that exposure to physiological and emotional stressors induces rapid hippocampal and cortical tau-P (for review, [42]). Accordingly, animal models of chronic stress employing overexpression of CRF and/or exposure to stressors are characterized by changes in synaptic structure, brain atrophy, neurodegeneration, hippocampal-dependent cognitive deficits, tau-P and detergent-soluble pools of phosphorylated tau with definable structure [52–59]. Mechanistic studies suggest that stress-induced tau-P can occur in the absence of an increase in stress steroids, but is dependent on CRF signaling through CRFR1 [55–58, 60].

Although the human and rodent in vivo data reviewed above suggest important mechanistic roles for CRF, CRFR1 and stress exposure in AD neuronal vulnerability and pathology, the actual cellular changes that lead to these reported detrimental effects have not been elucidated. Here we developed an in vitro stress platform to explore the effects of acute and chronic CRF exposure and tau-P on vital cellular processes such as axonal transport of growth factors and energetics.

## Materials and Methods

### Neuronal Cultures

Primary hippocampal neuronal cultures were generated from embryos at day 18.5 (E18.5) from timed-pregnant C57BL/6J mice (Charles River Labs) using established methods [61, 62]. Dissociated neurons were plated in poly-D-lysine coated 12-well culture plates at a density of 1–1.5 million cells per well for mass cultures, and 0.2 million cells per coverslip for immunocytochemistry. Plating density of cultures for all experiments were based on previous reports [63] and our internal testing/validation (data not shown). Plating media was replaced with maintenance media (Neurobasal, 1% Glutamax and 2% B27) 2 hours after plating. To maintain maximum viability, cells were maintained in culture for 7 days before experiments and two-thirds of the medium was replaced every other day. All experimental protocols involving the use of animals were approved by University of California, San Diego Institutional Animal Care and Use Committee.

### In vitro stress treatment

To establish a time course of CRF-induced tau phosphorylation, cells were treated with 1 μM (low) and 10 μM (high) CRF for 0, 0.5, 2, 4, 6, 8 and 24 hours (hr) (n = 3 each), and processed for Western blot as described below. CRF (Bachem) was reconstituted in 10% ethanol. Dosages of CRF treatment were based on previous reports [63] and our internal testing/validation (data not shown). Based on the results of the time course, 10 μM CRF for 0.5hr (acute stress) or 2hr (chronic stress) was selected for immunocytochemistry and axon transport experiments. To isolate the impact of CRF-induced tau-P on axonal transport, GSK activity was blocked by pretreating cultures with 25mM Lithium Chloride (Sigma Aldrich) for 30 minutes prior to adding CRF. Vehicle controls were run in parallel for all conditions.

### Axonal transport studies

To define the impact of CRF-induced tau-P on axon transport, we elected to examine axonal transport of two types of cargoes: 1) mitochondria that displays both anterograde and retrograde movement within axons [64] and 2) BDNF signaling in endosomes are initiated from axonal terminals and are retrogradely transported to the soma to deliver trophic support [61, 62].

To separate axons from cell bodies, 450 nm microfluidic chambers (Xona Microfluidics) were prepared using established methods [61, 62]. Axons were imaged using an inverted epifluorescent microscope equipped with an environmental chamber (37°C, 5% CO_2_) and a 100x oil objective lens (Leica DMI6300). A series of time-lapse images (2 frames/second) were collected using a CCD camera (Qimaging). Both DIC and fluorescent images were acquired to ensure consistent neuronal health before and after the acquisition of time-lapse images. NIH ImageJ and Leica Metamorph software were used to assemble time-lapse movies and to generate kymographs for quantitative analysis.

For mitochondria studies, cells were treated with 10 μM CRF and 2.5 μM Mitotracker (Life Technologies) for 30 minutes. Video image series of mitochondria in the distal axons were collected at 1 frame/sec for a total 60 sec [62]. For BDNF studies, maintenance media was replaced with Neurobasal media for one hour prior to treatment in order to deplete cells and axons of BDNF. 45 minutes prior to live imaging, 0.2 nM BDNF conjugated to QD655 (Life Technologies) was added to the distal axons.

### Western blot analysis

Hippocampal neurons plated in 12-well plates (as described above) were washed with cold phosphate buffered saline (PBS), collected and centrifuged at 1000 rcf for 5 minutes at 4°C. Resultant pellets were lysed in detergent-containing radioimmunoprecipitation assay (RIPA) buffer containing phosphatase inhibitors (1 μM okadaic acid, 1 mM Na_3_VO_4_) and a protease inhibitor cocktail (Thermo Scientific) as described [56, 57]. Western blot data was analyzed using NIH ImageJ software as described [56, 57].

### Immunocytochemistry

Hippocampal neurons were plated on poly-L-lysine coated coverslips. After treatment with CRF (10 μM for 2hr), cultures were washed with cold PBS, fixed with 4% paraformaldehyde for 10 min, quenched with 0.1 M ammonium chloride, and permeabilized with 0.1% PBS-triton for 10 minutes. Coverslips were blocked with 1% bovine or goat serum albumin in PBS for 30 minutes and then incubated in primary antibodies overnight at 4°C. Secondary antibody (Alexa 488, Life Technologies) was applied the next morning for 1hr at room temperature, followed by DAPI or Hoechst stain for 10 minutes, and then mounted for imaging. Images were acquired using a Leica DMI6000B inverted microscope with 20x objective lens by scanning the cells directly adjacent to the microgrooves. The threshold for all images was set equally using NIH ImageJ software and the “analyze particles” function was used to automatically count positive nuclei (Hoechst and pCREB experiments).

*Antibodies*. The well-characterized phospho-specific antibody, PHF-1, was used to detect tau-P at S^396/404^ (gift of Dr. P. Davies, Albert Einstein College of Medicine). PHF-1 was selected as a representative marker of stress-induced tau phosphorylation based on extensive characterization in our prior work [56–58, 60]. We previously validated PHF-1 for use in mouse tissue treated with alkaline phosphatase, which eliminated detectable PHF-1 labeling [58]. Changes in the GSK, implicated in the phosphorylation of tau at S^396/404^ was also assessed. Activated GSK-3β was detected using anti-GSK-pY^216^ (BD Bioscience), and inactive GSK-3β was assessed using anti-GSK-pS^9^ (Cell signaling) [65]. Antibodies targeting the C-terminus of β-actin (Sigma Aldrich) were used as a control for protein loading on Western blots. An antibody to pCREB antibody was used to target phosphorylated CREB at S^133^ (Cell Signaling).

### Statistical Analysis

Optical density readings from Western blots and data from axon transport experiments were analyzed by one or two way ANOVA using Prism 6 software (GraphPad, San Diego, CA). Data are expressed as mean ± SEM, normalized to β-actin loading.

## Results

### Timecourse of CRF-Induced Tau-P

**Fig 1:**
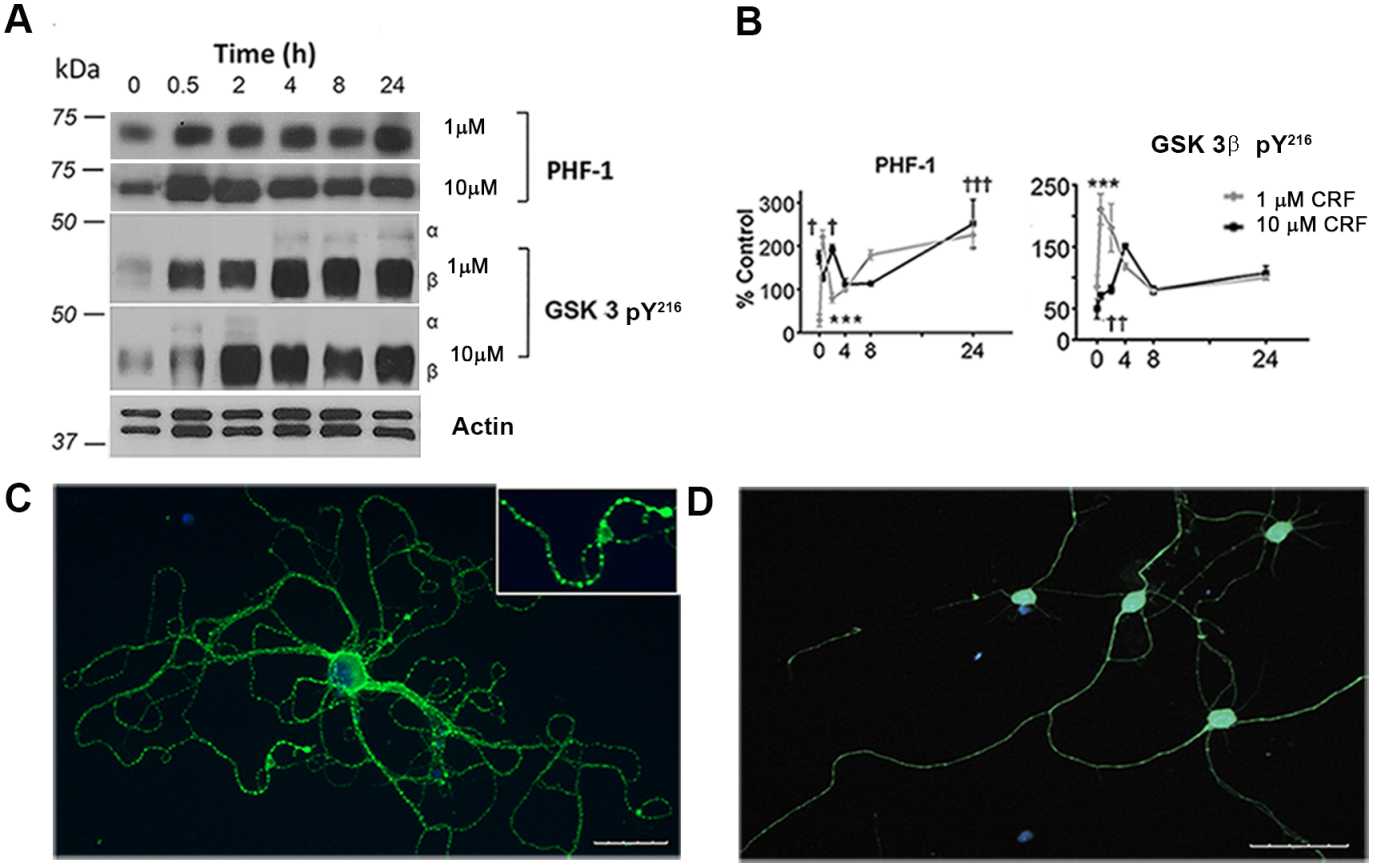
Stress-induced tau-P and kinase activation. **(A)** Western blot of PHF-1 and GSK-3 pY^216^ in cultured mouse hippocampal cells exposed to low or high concentrations of stress hormone CRF (1 μM or 10 μM) over a period of 0, 0.5, 2, 4, 8, or 24 hours with β-actin as a loading control. **(B)** Quantitative analysis of western blots (n = 3). Treatments differ significantly from controls; PHF-1 † (p = 0.01), ††† (p<0.001), *** (p<0.001); Active GSK 3β pY^216^ †† (p = 0.006), ††† (p<0.001), *** (p<0.001). **(C)** Immunostaining with PHF-1 (green) of neuronal treated with 10 μM CRF and **(D)** vehicle control cell nuclei with DAPI staining (blue).

To determine the consequences of stress-induced tau-P on cellular function, we first determined whether increased tau-P in response to CRF treatment was observable in our culture system. Western blot analysis was used to examine levels of tau-P at the AD-relevant C-terminal site (PHF-1) in extracts from hippocampal neuronal cultures after 0, 0.5, 2, 4, 8 or 24hr of 1 μm (low) or 10 μm (high) CRF treatment (Fig 1A and 1B). Compared to vehicle control (V), neurons treated with either 1 μm or 10 μm CRF exhibited a significant increase in tau-P immediately after treatment (0hr, p = 0.02), 2hr (p = 0.01) and through 24hr of treatment (p< 0.001). In terms of upstream kinase mediators, we observed parallel changes in activated GSK-3β (pY^216^), in concordance with the PHF-1 data. A significant increase was observed in active GSK-3β at the 2hr (p<0.001), 4hr (p = 0.01), 8hr and 24hr (each, p<0.001) timepoints with 10 μm CRF treatment. No change was observed in the inactive form of GSK-3β (pS^9^) for any timepoint after 1 μm or 10 μm CRF treatment (all p>0.05 compared to vehicle, data not shown). Because elevation in tau-P was stable overtime, the 10 μm CRF treatment and the 2hr timepoint were selected for immunohistochemistry, and both the 0.5hr and 2hr timepoints were selected to simulate acute and chronic stress, respectively, in axon transport experiments.

### Cellular Localization of Tau-P

Immunocytochemical methods were used for qualitative analysis of tau-P as a function of CRF treatment. Consistent with our biochemical data, vehicle-treated cultures showed low basal levels of PHF-1 labeling, which was prominently increased with CRF treatment. In vehicle treated cultures, we observed a relatively linear distribution of PHF-1 along neuron projections, consistent with basal levels of tau-P in neurons (Fig 1D). However, in CRF-treated cultures, we observed greatly increased intensity of PHF-1 staining and the presence of globular accumulations localized in neurites (Fig 1C, inset). These qualitative results suggest that CRF induces tau-P in vivo, which accumulates in dendrites and axons, and may cause blockages that are consistent with impaired axon transport.

### Impact of CRF-Induced Tau-P on BDNF Axonal Transport

**Fig 2:**
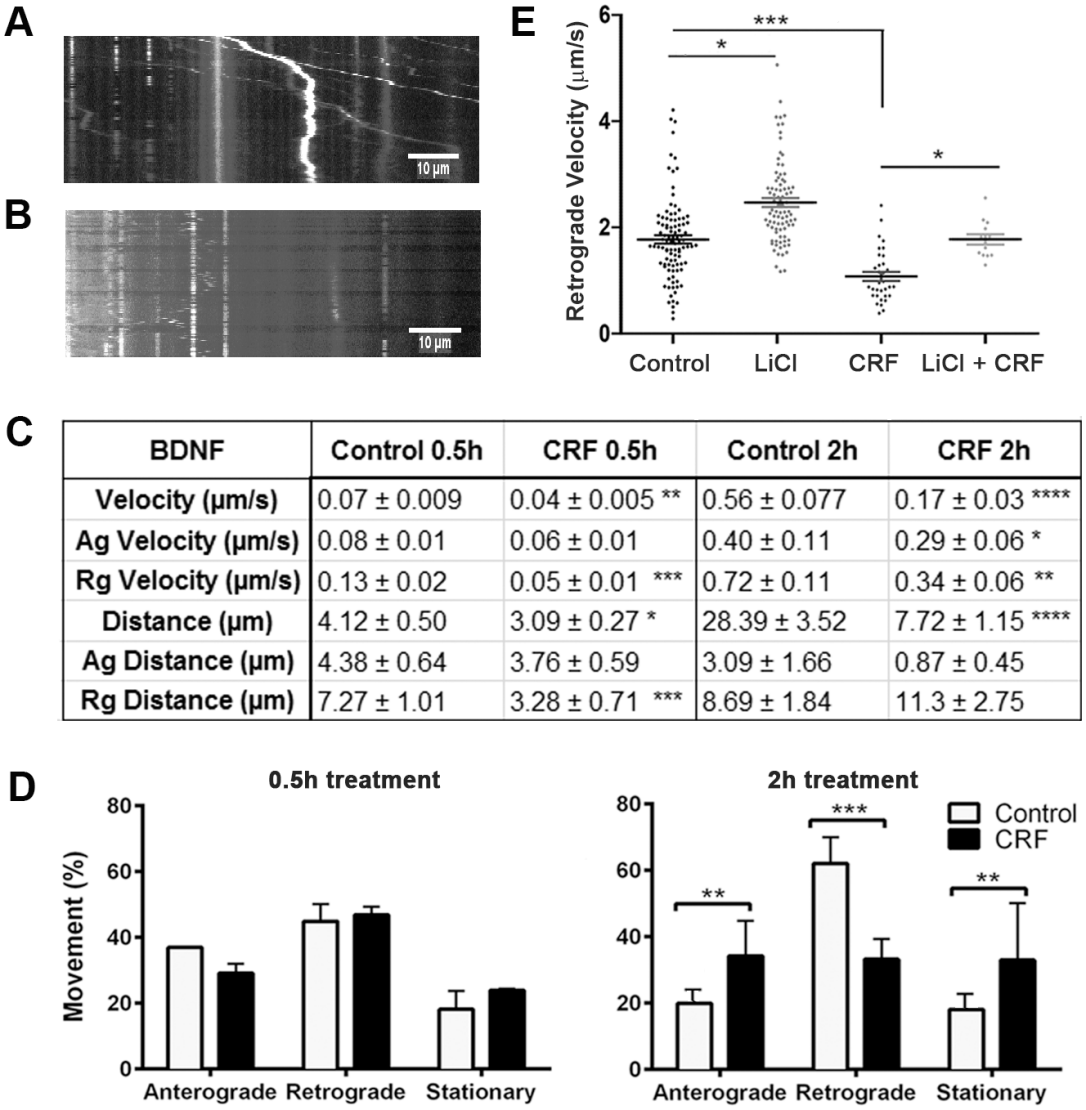
Impact of stress-induced tau-P on BDNF transport. Overall velocity of QD-BDNF was reduced in 0.5hr CRF cultures (overall velocity **p = 0.03, retrograde ***p = 0.0002) and 2hr CRF treatment (****p = 0.008); 2hr CRF treated cultures also exhibited reduced anterograde and retrograde velocity (*p = 0.03, **0.0002, respectively); Distance travelled was reduced in 0.5hr CRF treated cultures (overall *p = 0.04, retrograde ***p = 0.01) and 2hr CRF treatment (****p = 0.0001). All compared to vehicle treated controls; **(D)** Analysis of percent mitochondrial movement at 0.5hr and 2hr revealed no changes with 0.5hr CRF treatment (all p>0.05), though 2hr CRF treatment induced dramatic changes in anterograde (p = 0.001), retrograde (p = 0.0001) and stationary (p = 0.01) movement; **(E)** As seen in D, CRF treatment reduced retrograde velocity (***p = 0.02); 25mM LiCl treatment increased basal levels of retrograde velocity in vehicle control cultures (*p = 0.03) and prevented CRF-induced reductions in retrograde velocity (p>0.05, vehicle control compared to CRF+LiCl; CRF vs LiCl, *p = 0.03).

Microtubule-based axonal transport of organelles and other cargoes is a vital process for maintenance of neuronal structure and function, and synaptic plasticity [66]. Impairment or disruption of axonal transport has been proposed to be an early pathological feature of neurodegenerative diseases such as AD [67, 68]. Because BDNF plays a crucial role in neuronal development and neuronal plasticity, we examined the effects of CRF-induced tau-P on BDNF transport (Fig 2). Live imaging of Quantum-dot labeled BDNF was performed using our previously published protocols [61, 62] and kymographs were generated from time-lapse videos recorded from both vehicle and 10 μm CRF-treated neurons (Fig 2A and 2B). Measurements of distance travelled, velocity and directionality of movement relative to the cell body (anterograde, retrograde, or stationary). With acute CRF treatment (0.5hr timepoint), CRF-treated neurons exhibited significantly reduced overall and retrograde distance travelled of QD-BDNF compared to vehicle treated controls (p = 0.04 and p = 0.01, respectively) (Fig 2C). We also observed a significant reduction in overall and retrograde velocity (p = 0.03 and p = 0.0002, respectively) (Fig 2C). Conversely, no significant change in percent movement was observed with acute CRF treatment (all, p>0.05, Fig 2D). Similar to that seen with acute CRF treatment, chronic CRF treatment caused significant reductions in overall (p = 0.008), anterograde (p = 0.03) and retrograde (p = 0.0002) velocity and distance travelled (p = 0.0001) of QD-BDNF (Fig 2C, S1 Movie vs S2 Movie). Unlike that seen with acute CRF treatment, chronic CRF treatment led to large changes in transport direction relative to the cell body, with increased anterograde transport (p = 0.001), decreased retrograde transport (p = 0.0001), and increased stationary (p = 0.01) QD-BDNF (Fig 2D) compared to vehicle treated controls.

To directly isolate the mechanistic involvement of tau-P in observed axon transport deficits, cultures were incubated with 25mM lithium chloride (LiCl) alone and in combination with acute CRF treatment, and the movement of QD-BDNF was studied. Compared to vehicle control treated cells the LiCl treated cells showed a significant increase in retrograde velocity (p = 0.03) while CRF treatment significantly decreased retrograde velocity (p<0.0001) (Fig 2C and 2E). Co-treatment of CRF and LiCl prevented CRF-induced decrease in retrograde transport of QD-BDNF (p = 0.03) compared to cells treated with only CRF (Fig 2E). The retrograde velocity of QD-BDNF in cells treated with CRF and LiCl were not significantly different from vehicle control-treated cells (p>0.05). Collectively these results demonstrate that CRF-induced alterations in BDNF transport is dependent on tau-P.

### CRF treatment reduced pCREB activation in neuronal soma

**Fig 3:**
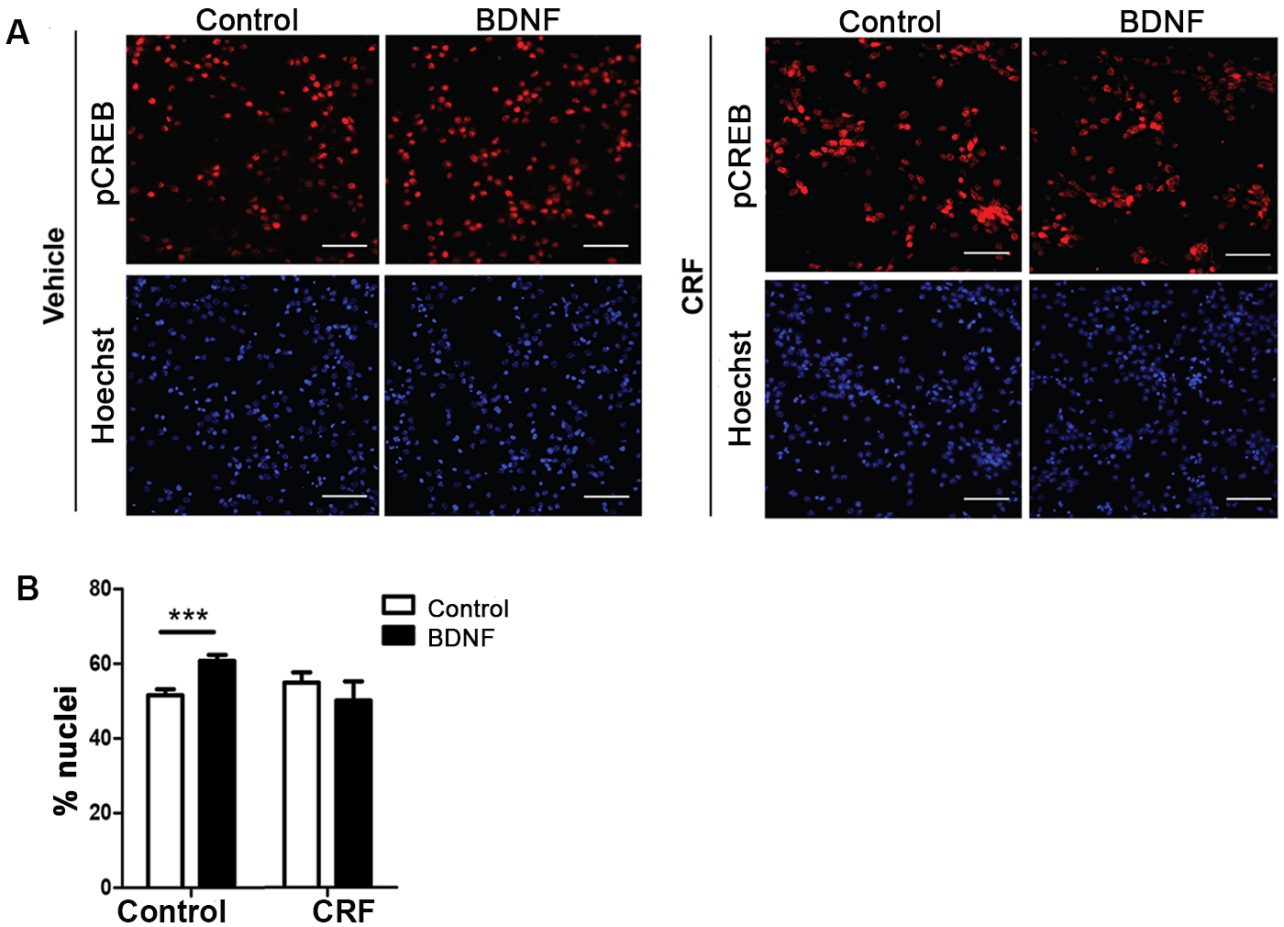
Activation of CREB Pathways. Neurons that were cultured in microfluidic chambers were treated with BDNF (50 ng/ml) for 0.5hr. Neurons were fixed and stained for pCREB and the nuclei were stained with Hoechst dye. **(A)** Representative images are shown (scale bar = 50 μM). **(B)** Quantitative analysis of the percentage of nuclei that were pCREB-positive (p = 0.005, n = 10 images).

One principal effector of retrograde axonal transport and signaling of BDNF is the activation of CREB (cAMP response element-binding protein) through its phosphorylation [62]. We predicted that impairment of BDNF trafficking, as elicited by CRF treatment, could also disrupt neuronal function by decreasing the activation state of CREB following axonal stimulation with BDNF. Our results demonstrate that vehicle-treated cells exhibit a significant increase (p = 0.0005) in the percent of nuclei positive for phosphorylated CREB in response to BDNF, as expected (Fig 3). However, neurons treated with CRF failed to show a response to axonal BDNF application (Fig 3). These results suggest that altered BDNF transport as a result of CRF-induced tau-P involves changes in pathways involved in activation/phosphorylation of CREB.

### Impact of CRF-Induced Tau-P on axonal movement of mitochondria

**Fig 4:**
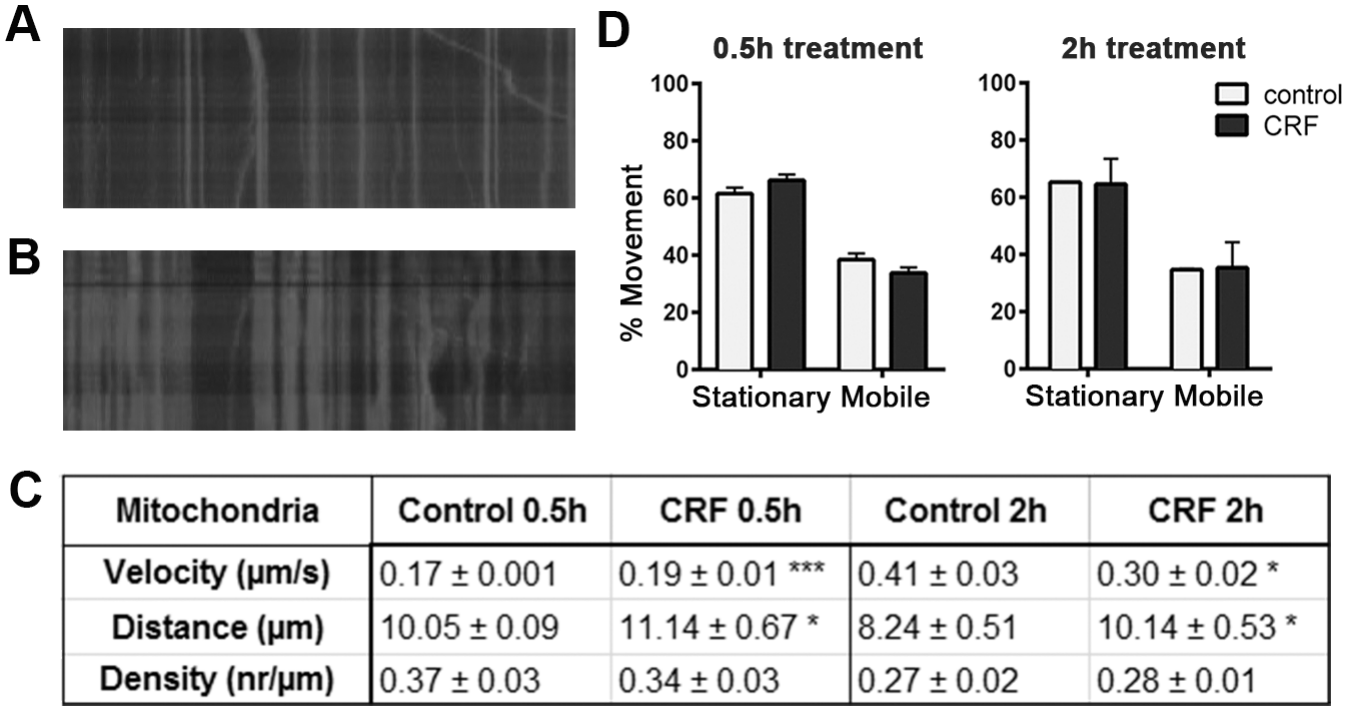
Impact of stress-induced tau-P on mitochondria transport. Kymographs showing axons of cultured mouse hippocampal cells after 2hr treatment; **(A)** Vehicle control. **(B)** CRF. **(C)** Table of quantitative analysis of fluorescent live images acquired from axons of cultured mouse hippocampal cells treated with 10 μM CRF compared to controls; Average velocity of mitochondria 0.5hr (***p = 0.0002), 2hr (*p = 0.02); Distance travelled 0.5hr (*p = 0.04), 2h (*p = 0.01); Density of mitochondria after 0.5hr and 2hr. **(D)** Percent of mitochondrial movement at 0.5hr and 2hr.

We next explored the effects of CRF-induced tau-P on the movement of mitochondria using Mitotracker (Fig 4). Live imaging was performed to capture time-lapse images of mitochondrial movement. Kymographs were generated from these time-lapse images in order to analyze changes in the axonal transport of mitochondria (Fig 4A and 4B). After acute CRF treatment, mitochondria showed a significant increase in the overall velocity (p = 0.0002) and distance travelled (p = 0.04) compared to controls (Fig 4C). However, with chronic CRF treatment, mitochondrial velocity decreased significantly (p = 0.02), while a significant increase in the distance traveled by mitochondria (p = 0.01) was once again observed (Fig 4C and S3 Movie vs S4 Movie). No significant change in either the overall density or direction of movement were observed (Fig 4C). In addition, treatment with acute or chronic CRF did not significantly change the ratio of moving versus stationary mitochondria as compared to vehicle controls (Fig 4D). These results suggest that, unlike that seen for BDNF (Fig 2), mitochondrial transport may be sensitive to the effects of acute vs chronic stress. Acute exposure to CRF induces increased mitochondrial transport velocity, while chronic exposure lead to a reduction in mitochondrial velocity but an increase in distance travelled.

As performed in BDNF experiments, parallel cultures where incubated with LiCl during acute CRF treatment. As seen for BDNF, the mitochondrial velocity was significantly increased in LiCl treated cells (p = 0.03) compared to vehicle control cells. Accordingly, combined treatment of CRF and LiCl resulted in a significant increase in velocity and distance travelled of mitochondria (p = 0.03, each) compared cells treated with only CRF (data not shown).

## Discussion

In this study, we developed an in vitro model system to explore the functional consequences of acute and chronic stress-induced tau-P on neuronal function. We find that CRF treatment induces tau-P in a manner similar to that reported with stress models in vivo, and demonstrate that the axon transport deficits observed in our model involves CREB phosphorylation and is reliant on tau-P via GSK activation. Collectively, our data underscore the importance of CRF as a neuromodulator/neurotransmitter and provides a mechanistic underpinning for stress-induced tau-P and work that implicates CRF as a target for therapeutic intervention in AD.

### Axon transport of Mitochondria and BDNF

In our in vivo work, we observed phospho-tau positive beading in varicosities along axons in stressed mice (RA Rissman, unpublished observations) similar to dystrophic neurite pathology in AD, suggesting structural alterations (i.e. blockages) and transport dysfunction [19, 20, 69]. A major hypothesis in the AD field suggests that defects in transport of important vesicles contributes to neurodegeneration [67, 68]. Phosphorylated tau accumulations have been reported to block transport of organelles, mitochondria, and other vesicles in neurons [70, 71], consistent with our current results. To probe the functional relevance of CRF-induced tau-P, we monitored changes in axon transport of two critical processes, energetics (i.e. mitochondria) and a major neuronal growth factor, BDNF, as a function of CRF treatment.

Mitochondria are concentrated in areas of high ATP demand, such as pre and post synaptic terminals, and neuronal growth cones. With the dynamic nature of neurons, mitochondria must be able to rapidly redistribute to different areas in order to meet increased energy demands to support mobilization of synaptic vesicles, actin assembly and disassembly, and generation of membrane potentials. Mitochondria are transported to activated synapses in response to ATP levels: velocity increases in areas of high ATP and decrease in areas of depleted ATP [70]. As observed in our study, short-term (i.e. 0.5hr) CRF exposure increased velocity of mitochondria, consistent with neurons eliciting an adaptive response of mobilizing energy resources to address to the challenge. We hypothesize that mitochondrial movement increased due to higher energy demands in the cell body and synaptic terminal during stress. Conversely, we observed a reduction in mitochondrial velocity with chronic CRF treatment, which we hypothesize may be due to increased accumulation of tau-Pand axonal blockage [19]. Accordingly, models of tau overexpression are characterized by blockages of microtubule tracks and reduction of transport of vesicles and cell organelles [19]. Blockage of vesicles and cell organelles can lead to depletion of crucial supplies required for cellular processes. Although not examined in this study, tau-P has also shown to inhibit movement of motor proteins [72, 73], which may also have a substantial impact on changes in mitochondrial movement. We hypothesize that the physical blockage of axons by tau-P leads to dysfunction of motor proteins and therefore a decrease in mitochondrial velocity, depletion of local ATP, oxidative stress, and interruption of neuronal function.

BDNF is an important neurotropic factor critical to neuronal survival, development, network connectivity, and formation of synaptic connections, and is found in high concentration in the cerebral cortex and hippocampus [74]. BDNF has neuroprotective benefits, as demonstrated in many animal models of AD [75]. Chronic stress exposure in vivo decreases BDNF expression levels in the dentate gyrus and hippocampus [24, 76, 77]. Tau pathology is a common component of chronic stress and AD, but it is unclear what effects these have on BDNF transport. Efficient retrograde axonal trafficking of BDNF is essential to properly initiate and sustain signaling both locally in the axon and at the soma. Previous studies suggest that treatment of neurons with other AD-related proteins, such as Aβ, cause deficits in BDNF trafficking [78]. We therefore investigated the effects of CRF-induced tau-P on BDNF transport. Using the same parameters as the mitochondria study, changes in mitochondrial movement were apparent with both acute and chronic CRF treatment. However, unlike that seen in our mitochondria experiments, we did not observe acute stress-like increases in transport of BDNF with CRF treatment—both acute and chronic treatment caused reduction in velocity and distance travelled of BDNF, suggesting that cellular energetics may be a priority to cells undergoing stress as compared to growth factor transport. Although activation of the stress axis is a crucial response to challenge, our findings suggest that under chronic/repeated stress, neurons may be starved of growth factors. This response may be restricted to stressful stimuli, as studies of “good stressors” such voluntary exercise demonstrate increases in BDNF bioavailability [79, 80].

Activation of the transcription factor, cAMP response element-binding protein (CREB) has been observed in the nuclei of neurons after axonal treatment with BDNF [81]. CREB activation is responsible for the expression of several genes including those involved in maintenance of neuronal morphology and dendritic development [82]. Importantly, CREB is downstream of the mitogen-activated protein kinase (MAPK) pathway, one of the signaling pathways induced by BDNF stimulation. Activation of CREB by BDNF and the ensuing retrograde axonal transmission of CREB to the soma is an integral part of the signaling pathways of BDNF, which provides critical trophic support to neurons. Blockade or inhibition of these processes often results in axonal degeneration and neuronal atrophy [61, 62]. Our current studies reveal that stress hormones such as CRF, through induction of tau-P, may exert a similar adversary effect on axonal and neuronal function.

## Conclusions

Our study demonstrates that CRF induces tau-P in a manner consistent with our in vivo studies [56–58]. In these in vivo studies, we found that chronic stress or CRF overexpression caused sequestration of tau aggregates into detergent-soluble cellular fractions [56, 57]. The data presented here provide functional information regarding the consequences that CRF and tau-P may have on neuronal function. CRF-induced tau-P interfered with axon transport of mitochondria and BDNF, which we hypothesize can lead to impairments in transportation of cargoes, depleted trophic factor supply, and oxidative stress that may contribute to AD pathogenesis. Continued exploration of the circuits and mechanism of stress-induced tau-P is central to further uncover the relationship between stress and AD tau pathology.

## Supporting Information

S1 Movie BDNF movement under control/vehicle conditions. Time lapsed video of QD-BDNF movement in neuronal axons under vehicle conditions.
(MP4)

S2 Movie BDNF movement with CRF treatment. Time lapsed video demonstrating altered movement of QD-BDNF in neuronal axons after 2hr CRF treatment.
(MP4)

S3 Movie Mitochondrial movement under control/vehicle conditions. Time lapsed video of mitochondrial movement in neuronal axons under vehicle conditions.
(MP4)

S4 Movie Mitochondrial movement with CRF treatment. Time lapsed video demonstrating altered movement of mitochondria in neuronal axons after 2hr CRF treatment.
(MP4)
